# 10-year follow-up of the Super-Seniors Study: compression of morbidity and genetic factors

**DOI:** 10.1186/s12877-019-1080-8

**Published:** 2019-02-28

**Authors:** Lauren C. Tindale, Diane Salema, Angela R. Brooks-Wilson

**Affiliations:** 1 Canada’s Michael Smith Genome Sciences Centre, BC Cancer Research Centre, 675 West 10th Ave, Vancouver, BC V5Z 1L3 Canada; 2 Biomedical Physiology and Kinesiology, Simon Fraser University, Burnaby, BC Canada

**Keywords:** Oldest-old, Centenarians, Super-seniors, Healthy aging, Longevity

## Abstract

**Background:**

Super-Seniors are healthy, long-lived individuals who were recruited at age 85 years or older with no history of cancer, cardiovascular disease, diabetes, dementia, or major pulmonary disease. In a 10-year follow-up, we aimed to determine whether surviving Super-Seniors showed compression of morbidity, and to test whether the allele frequencies of longevity-associated variants in *APOE* and *FOXO3* were more extreme in such long-term survivors.

**Methods:**

Super-Seniors who survived and were contactable were re-interviewed 10 years after initial characterization. Health and lifestyle were characterized via questionnaire. Geriatric tests including the Timed Up and Go (TUG), Geriatric Depression Scale (GDS), Instrumental Activities of Daily Living (IADL) and the Mini-Mental State Exam (MMSE) were administered, and data were compared to results from on average 10 years earlier. As well, genotype and allele frequencies for SNPs rs7412 and rs429358 in *APOE*, and rs2802292 in *FOXO3* were compared to the frequencies in the original collection of Super-Seniors and mid-life controls.

**Results:**

Of the 480 Super-Seniors recruited from 2004 to 2007, 13 were alive, contactable, and consented to re-interview (mean age = 100.1 ± 3.3). Eight of these 13 participants (62%) still met Super-Senior health criteria. Diseases that occurred in late life were cardiovascular (5 of 13; 38%) and lung disease (1 of 13; 8%). MMSE and IADL scores declined in the decade between interviews, and GDS and TUG scores increased. The surviving group of centenarians had a higher frequency of *APOE* and *FOXO3* longevity-associated variants even when compared to the original long-lived Super-Senior cohort.

**Conclusions:**

Although physical and mental decline occurred in the decade between interviews, the majority of Super-Seniors re-interviewed still met the original health criteria. These observations are consistent with reports of compression of morbidity at extreme ages, particularly in centenarians. The increased frequency of longevity- associated variants in this small group of survivors is consistent with studies that reported genetics as a larger contributor to longevity in older age groups.

**Electronic supplementary material:**

The online version of this article (10.1186/s12877-019-1080-8) contains supplementary material, which is available to authorized users.

## Background

Maintaining health while aging is important both for individual quality of life as well as costs to health care systems. Compression of morbidity refers to a shorter time between onset of disability and death, and was originally postulated to be due to chronic conditions having a greater capacity to be delayed than survival has to be increased [1]. A correlation between survival age and decreased morbidity has been observed, where older age groups (100–104 years, 105–109 years, and 110–119 years) experienced progressively delayed onset of disease and physical or cognitive impairment [2].

While the overall incidence of chronic conditions has been increasing in recent decades [3, 4], compression of morbidity has been observed in long-lived individuals [5–8]. As studies of younger groups (e.g., 51–61 years) [4] did not reveal compression of morbidity, the reduction in years of disease may be limited to the high end of the human life span.

The Super-Seniors were collected as a phenotypically healthy oldest-old group in which to study genetic factors associated with healthy aging [9, 10]. Super-Seniors are individuals aged 85 years and older who reported never being diagnosed with cancer, cardiovascular disease (CVD), diabetes, dementia, or major pulmonary disease. Study controls are a population-based comparison group of mid-life individuals who resemble a genetic proxy group for the birth cohort of the Super-Seniors [10]. The initial collection and characterization of 480 Super-Seniors took place from 2004 to 2007 (Phase 1), with an additional and ongoing collection initiated in 2014 (Phase 2).

Approximately 10 years after the Phase 1 collection of Super-Seniors, we attempted to re-contact and re-interview surviving Super-Seniors. Participants still living would be in their late nineties or 100 or more, and we hypothesized that such individuals would have retained much of their health due to compression of morbidity.

## Methods

Research ethics board approval was received from the joint Clinical Research Ethics Board (REB) of BC Cancer and the University of British Columbia and from the REB of Simon Fraser University. All subjects gave written informed consent.

Phase 1 collection of Super-Seniors in 2004–2007 [10] did not include plans to follow participants longitudinally, so in 2010, Super-Seniors were mailed a letter requesting permission to re-contact them for future research. In 2016–17, contactable and interested Super-Seniors were invited for re-interview (Additional file 1: Figure S1).

Super-Seniors were visited in their homes where they were asked personal and family medical history questions, and asked to perform geriatric tests as per their first interview. The Mini-Mental State Exam (MMSE) [11], Instrumental Activities of Daily Living (IADL) [12], Geriatric Depression Scale (GDS) [13], and Timed Up and Go (TUG) [14] were measured and compared to data from Phase 1 Super-Seniors collected approximately 10 years previously. Differences in scores were analyzed in one-tailed paired t-tests with the expectation that MMSE and IADL score would decrease with age, and that GDS and TUG scores would increase. BMI, HR, and BP were compared using two-tailed paired t-tests.

Genotype and allele frequencies for SNPs rs7412 and rs429358 in *APOE*, and rs2802292 in *FOXO3* were compared to the frequencies in the original Phase 1 Super-Seniors and mid-life controls. Genotypes for these select variants were extracted from data previously described [15]. Statistical analysis was done using R 3.2.2 and JMP 13.

## Results

In 2010, we mailed 480 Super-Seniors and an additional 17 borderline phenotype individuals from Phase 1, excluding 9 for whom the study had been notified of their death. Twenty-six were reported deceased by relatives, 30 declined (5/30 indicated they were “too sick”), 92 were unable to be contacted (mail was returned to sender), 246 did not respond, and 94 agreed to be re-contacted (Additional file 1).

In 2016, we searched for online obituaries and notices of death, and sent another letter requesting permission for re-contact to 139 previous non-responders. By 2016, an additional 107 Super-Seniors were determined to be deceased through online records or reported to the study by family members. Of the 138 Super-Seniors mailed in 2016, 8 were reported deceased, 7 declined (3/7 indicated they were “too sick”), 26 were unable to be contacted, 89 did not respond, and 9 agreed to be re-contacted.

Twenty-eight Super-Seniors were invited for interview. Of those, 13 accepted and completed the re-interview; 2 declined, 10 could not be reached, 1 died, and 2 were reported by relatives to be too ill to be interviewed. The 13 Super-Seniors re-interviewed in 2017 included 10 women and 3 men aged 96–106 (mean = 100.1, SD = 3.3), who were all of European ancestry.

The two Super-Seniors who were too ill to be interviewed included a 100-year-old woman reported by a family member to be confused much of the time, not doing very well, and feeling sick and tired. The other was a 106-year-old woman reported by a family member to be bedridden and mostly non-responsive. Deterioration reportedly took place around age 100–101 when she began suffering episodes of dementia. Other than dementia, she had no health problems and was very mobile until age 103.

Re-interviews took place 9.3–12.1 years (mean = 10.9, SD = 0.9) after the initial interviews (Additional file 2). Of the 13 Super-Seniors who were re-interviewed, 8 still met the health criteria for being a Super-Senior, 5 women and all 3 of the men. Of the 5 Super-Seniors who did not meet the criteria for enrollment at the time of their re-interview in 2017, all had developed CVD: two had strokes (at ages 97 and 100, respectively), two had heart conditions (mitral valve issue [age unknown] and pacemaker [at age 99]), and one who was interviewed at age 96 had a minor heart attack, minor strokes, and COPD.

Of the 13 Super-Seniors re-interviewed, 6 were never smokers and the remaining 7 had quit. The quitters smoked between 5 and 51 years (mean = 29.7, SD = 16.0). Of note, the individual who had COPD was a housewife who never smoked.

**Fig. 1:**
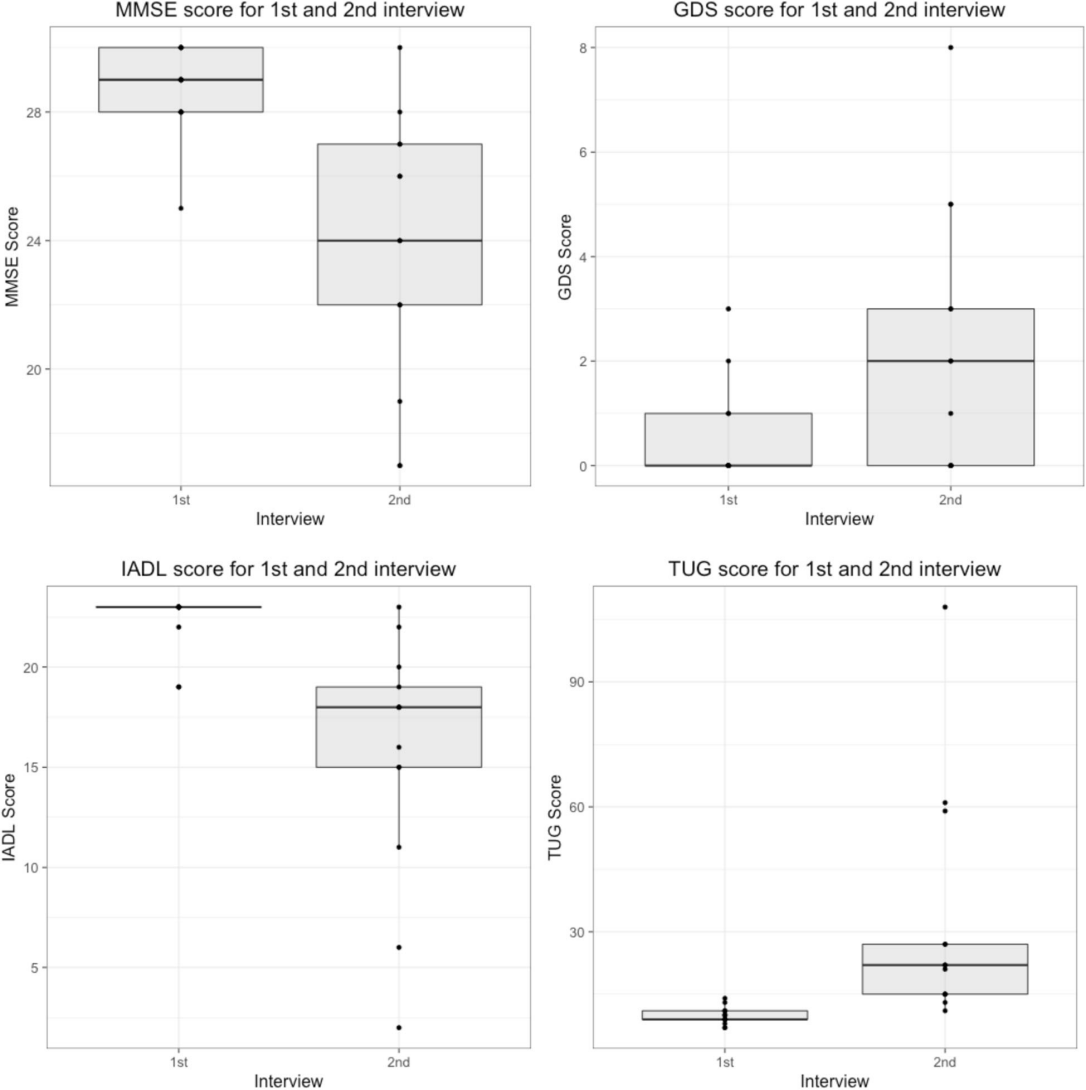
Super-Senior geriatric test scores at the first and second interviews approximately 10 years apart. MMSE = Mini-Mental State Exam, GDS = Geriatric Depression Scale, IADL = Instrumental Activities of Daily Living, TUG = Timed Up and Go

Descriptive statistics and geriatric test scores are shown in Table 1 and Fig. 1. MMSE mean scores declined from 28.7 (SD = 1.4) to 23.8 (SD = 4.2) points out of a possible 30, a mean decline of 4.9 points (t = − 4.5, *P* = 0.00036). IADL scores declined from 22.3 (SD = 1.5) to 15.6 (SD = 6.1) out of a possible 23 points, a mean decline of 6.7 points (t = − 3.7, *P* = 0.0016). GDS scores increased from 0.5 (SD = 1.0) to 2.2 (SD = 2.5), a mean difference of 1.7 points (t = 2.6, *P* = 0.011). TUG scores increased from 9.8 (SD = 2.1) to 32.0 (28.0) seconds, a mean difference of 22.2 s (t = 2.9, *P* = 0.0070). Among the 9 individuals who did not use a walker as an aid, the mean TUG time was 17.9 s (SD = 5.3).

**Table 1:** Characteristics of Super-Seniors at two interviews approximately 10 years apart

	Super-Seniors Study	Re-contacts		*P*-value
		First Interview	Second Interview	1st and 2nd interviews
Year	2004–2007	2004–2007	2016–2017	
N	480	13		
Male	155	3		
Female	325	10		
Age mean (SD) years	88.5 (2.9)	89.3 (2.7)	100.1 (3.3)	
Age range	85–105	85–94	96–106	
Years between interviews (SD)	–	10.9 (0.9)		
BMI mean (SD) kg/m^2^	24.5 (3.9)	25.8 (4.0)	23.4 (3.9)^a^	0.071
BP mean systolic (SD) mmHg	152 (21)^a^	142 (16)^a^	120 (35)	0.077
BP mean diastolic (SD) mmHg	78 (11)^a^	72 (9)^a^	72 (10)	0.980
Heart rate (SD) beats per min	70 (11)^a^	67 (9)^a^	74 (11)	0.120
MMSE mean (SD)	28.3 (1.7)	28.7 (1.4)	23.8 (4.2)	< 0.001
IADL mean (SD)	21.4 (3.5)	22.3 (1.5)	15.6 (6.1)	0.002
GDS mean (SD)	1.5 (1.8)	0.5 (1.0)	2.2 (2.5)	0.011
TUG mean (SD) seconds	12.3 (4.3)	9.8 (2.1)	32.0 (28.0)	0.007

^a^Blood pressure and heart rate were not available for all initial interviews. Super-Seniors Study blood pressure *n* = 298, heart rate *n* = 290. Re-contacts first interview blood pressure *n* = 12, heart rate *n* = 12. Re-contacts second interview BMI *n* = 12

There was no significant difference in BMI, heart rate, or blood pressure (BP) between the two interviews. Eight participants were taking BP medication at the time of both interviews, 4 were not taking any BP medication at either interview, and one man had discontinued taking BP medication by the time of the second interview.

Genotyping in re-interviewed Super-Seniors was not available for one participant in *APOE* and two participants in *FOXO3*. Genotype counts and minor allele frequency (MAF) values are shown in Table 2. Genotyping of Phase 1 Super-Seniors and controls was used for comparison [15].

**Table 2:** Genotype comparison between Super-Senior survivors, and the original Phase 1 collection of Super-Seniors and controls

	Re-interviewed Super-Seniors (*n* = 13)		All Super-Seniors (*n* = 466)		Controls (*n* = 421)	
	Genotypes	MAF	Genotypes	MAF	Genotypes	MAF
*APOE* rs7412	TT × 1 TC × 1 CC × 10	0.125	TT × 6 TC × 69 CC × 363	0.092	TT × 3 TC × 57 CC × 352	0.076
*APOE* rs429358	CC × 0 CT × 2 TT × 10	0.083	CC × 4 CT × 84 TT × 350	0.105	CC × 10 CT × 110 TT × 293	0.157
*FOXO3* rs2802292	GG × 0 GT × 9 TT × 2	0.409	GG × 55 GT × 226 TT × 162	0.379	GG × 47 GT × 189 TT × 166	0.352

*MAF* minor allele frequency

## Discussion

We attempted to re-contact Super-Seniors 9–12 years after they were enrolled. As expected for an older group, even one as healthy as the Super-Seniors, most participants had passed away within this time frame. We could confirm that at least 17 of the original 480 Super-Seniors were still living.

Eight of the 13 individuals re-interviewed in 2017 (54%) still met the criteria for being a Super-Senior; among the individuals who no longer met the Super-Senior criteria, most had developed their health problems in the previous 3 years. The most common diseases in the group were CVD (5 of 13; 38%), and lung disease (1 of 13; 8%). According to the US National Center for Health Statistics from 2012 to 2013, 29.8% of Americans aged 65 years and older reported having heart disease, and 18.4% reported having cancer [3]. As well, in a cohort of men born in 1913 and followed to age 100, CVD was the most common cause of death after the 80 years of age [16].

Although we cannot compare relative morbidity time between groups, our observations that 8/13 of the survivors were still “Super”, and that the 5/13 with chronic diseases developed them in their late 90s or early 100 s, these observations suggest that compression of morbidity is occurring in these individuals. Compression of morbidity has been suggested in other studies of long-lived people including the New England Centenarian Study, where they found that between nonagenarians, centenarians, semicentenarians (105–109 years), and supercentenarians (110–119 years), there was a later onset of major age-related diseases as the age group increased [2]. As well, among 15 Okinawa supercentenarians (age at death 110–112) it was found that they had delayed clinically apparent diseases until very late in life, with 83% not reporting clinically apparent disease until 105 years or later [17].

A German health insurance study also found that there was a lower prevalence of comorbidities among those who died as centenarians than those who died in their 80s [8]. Likewise, while 8 Super-Seniors were still disease free, 5 had one disease, and only one individual had co-morbidities.

We compared the current geriatric test scores of the Super-Seniors to their initial interview scores. MMSE and IADL scores declined in the decade between interviews, while GDS and TUG scores increased. For all four tests, the standard deviation increased from the first to second interviews indicating that there was a wider range of physical and cognitive function as the surviving participants aged.

Mean MMSE scores decreased from 28.7 to 23.8 points. Although this is a test of cognitive function, at least a portion of the decreased score was due to visual and/or hearing impairment, which affected the participant’s ability to answer some questions. At the time of the second interview, two individuals were legally blind, another was unable to see the images, and three were hard of hearing. Vision impairment for these individuals was not noted at the time of the first interview. It is worth noting however, that the one perfect score among the re-interviews was by a 102-year-old woman with macular degeneration. The traditional cut-off score for being “normal” and without cognitive impairment is 24, however a study of highly educated Caucasians found that a cut-off of 24 resulted in a moderate sensitivity (0.66) and very high specificity (0.99), whereas a cut-off score of 27 achieved a balance between sensitivity (0.89) and specificity (0.91) [18]. At the second interview, the Super-Seniors mean score almost meets the traditional 24-point cut-off. Individually however, the range of scores decreased from 25 to 30 points to 17–30 points indicating that at while some Super-Seniors have retained their cognitive function, others are experiencing increasing impairment.

All geriatric scores declined with age, with the second interview showing that the Super-Seniors had a mean higher depression score, decreased mobility, and were performing fewer daily tasks as measured by the IADL. Their GDS score, however, was still a mean of 2.2 points, below the value of ≥5 that has been indicated as an appropriate cut-off score for depression [19]. As well, although the TUG time increased from a mean of 9.8 to 32.0 s, 4 individuals used a walker as a mobility aid and one used a cane. The mean TUG time for individuals without a walker was 17.9 s. Although there is no specific standard for TUG time, it has been found that physical fitness indicators such as the TUG are associated with successful aging [20]. It has also been suggested that among adults 65 years and older with a similar disease burden, those who were more physically vigorous experienced compression of morbidity and lived longer [21]. A decreased IADL score indicates that they are performing fewer tasks independently; however, and in a study of individuals 90 years and older, regularly needing help was found to be a significant predictor of mortality [22].

The Okinawa centenarian study found that both BMI and BP decreased with age as individuals transitioned from centenarians to supercentenarians [17]. Longitudinally, when they followed individuals from age groups 99–103 years, to 104–107 years, to 108–111 years, their BMI decreased from 21.47, to 18.81, to 17.43, respectively. As well, their BP (systolic/diastolic) decreased from 142/74 mmHg, to 128/70 mmHg, to 119/64 mmHg. Similarly, following the Super-Seniors from ages 85–94 years, to 96–106 years, we saw a non-significant decrease in BMI from 25.8 (SD = 4.0) to 23.4 (SD = 3.9). Systolic BP of the Super-Seniors decreased from 142 mmHg (SD = 16) to 120 mmHg (SD = 35) and diastolic BP remained the same at 72 mmHg (SD = 9) and 72 mmHg (SD = 10). Interestingly, the re-interviewed Super-Seniors had lower values at their initial interviews for both their systolic and diastolic BP than the mean of the overall Phase 1 Super-Seniors cohort, 152/78 mmHg (Table 1). Both the values and the decline exhibited in Super-Seniors are consistent with what was observed in the Okinawa study.

*APOE* and *FOXO3* are the two loci most consistently associated with longevity, reviewed by: [23–25]. *APOE* has been found in genome-wide scans and cohort studies of longevity; the minor allele of rs7412 is sometimes associated with longevity, and the minor allele of rs429358 is reliably associated with increased mortality [26–31]. Together these two variants determine *APOE*2/3/4 haplotypes. At rs7412, the re-interviewed Super-Seniors had a MAF 0.125, compared to MAF 0.092 in all Phase 1 Super-Seniors, and MAF 0.076 in mid-life controls. At rs429358, the re-interviewed Super-Seniors had MAF 0.083, compared to 0.105 in all Super-Seniors, and 0.157 in controls. This demonstrates that among these elite survivors, Super-Seniors who survived another ~ 10 years, there appears to be a slightly higher frequency of the favorable longevity allele of rs7412, and a lower frequency of the deleterious mortality and AD-related allele of rs429358, even when compared to the original group of Phase 1 Super-Seniors.

In *FOXO3,* the G allele of rs2802292 has been associated with longevity [32]. 81.2% re-interviewed Super-Seniors carried at least one G allele, compared to 63.4% in all Phase 1 Super-Seniors and 58.7% of controls. Previously we did not find an association of rs2802292 with the Super-Senior phenotype, compared to mid-life controls, but we do see an apparent enrichment among this small group of survivors. This possible association may be stronger at more advanced ages, or it could be an artifact of the very small sample size.

It is important to note that this study is limited by very small sample size. Observations made in this small group of individuals may not be typical of centenarians in general. Partially contributing to this low number, however, is the fact that the majority of Super-Seniors were either not contactable or did not respond and, while most will have passed away, we do not know the exact number. As well, although the average age at recruitment was 88.5 years, some Super-Seniors were already in or near their 100 s at the time of initial recruitment and would therefore be less likely to be alive 10 years later. Given that the average lifespan for the Super-Seniors birth cohort (mean birth year 1916) was approximately 57 years, the 13 Super-Seniors who were re-interviewed represent a small but elite group who delayed disease onset until very late in life, and possibly exhibit compression of morbidity.

## Conclusions

Although physical and mental decline occurred in the decade between interviews, the majority of Super-Seniors re-interviewed still met the original health criteria. These observations are consistent with reports of compression of morbidity at extreme ages, particularly in centenarians [5, 7, 8]. The increased frequency of longevity-associated variants in this small group of survivors is consistent with studies that reported genetics as a larger contributor to longevity in older age groups [33, 34].

## Additional files

Additional file 1: Follow-up of participants in the Super-Seniors Study flow chart. (PDF 39 kb)

Additional file 2: Study dataset. (XLSX 38 kb)

## Abbreviations

BMI: Body mass index
BP: Blood pressure
COPD: Chronic obstructive pulmonary disease
CVD: Cardiovascular disease
GDS: Geriatric Depression Scale
IADL: Instrumental Activities of Daily Living
MAF: Minor allele frequency
MMSE: Mini-Mental State Exam
REB: Research ethics board
SD: Standard deviation
SNP: Single nucleotide polymorphism
TUG: Timed up and go
