# Supplementary figures and images for: 10-year follow-up of the Super-Seniors Study: compression of morbidity and genetic factors
Source: BMC Geriatr. 2019 Feb 28;19:58. doi: 10.1186/s12877-019-1080-8 (PMC6394013; doi:10.1186/s12877-019-1080-8)

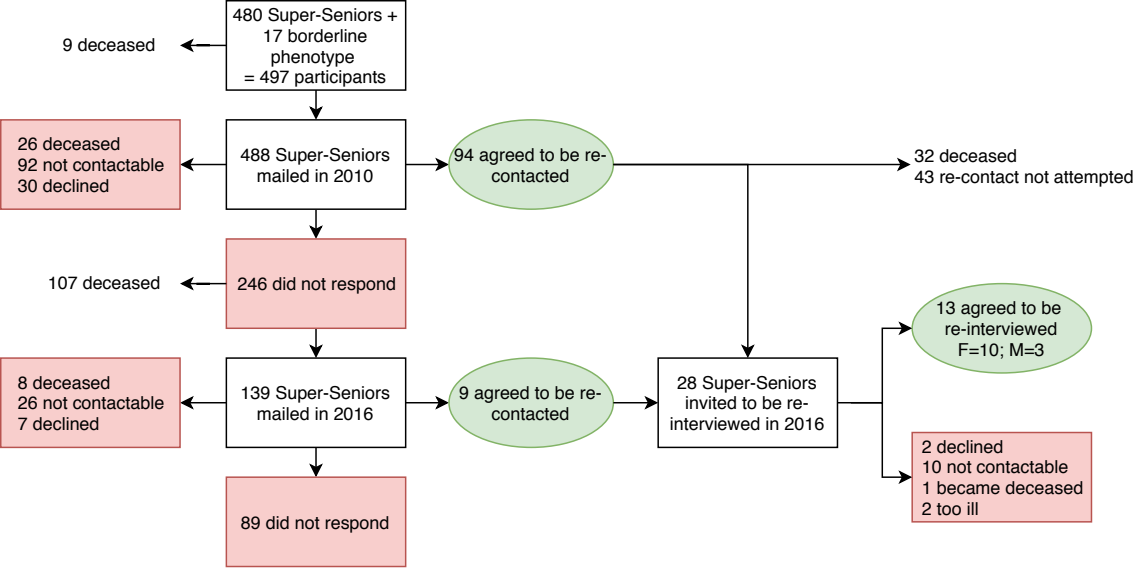

Supplement: Supplementary file 1 — Follow-up of participants in the Super-Seniors Study flow chart. (PDF 39 kb) [file 12877_2019_1080_MOESM1_ESM.pdf]
